# The Effect of Composition on the Properties and Application of CuO-NiO Nanocomposites Synthesized Using a Saponin-Green/Microwave-Assisted Hydrothermal Method

**DOI:** 10.3390/ijms25074119

**Published:** 2024-04-08

**Authors:** Amnah Al-Yunus, Wafa Al-Arjan, Hassan Traboulsi, Manal Hessien

**Affiliations:** 1 Department of Chemistry, College of Science, King Faisal University, Al Ahsa 31982, Saudi Arabia; 2 Department of Chemistry, Champlain College, 900 Riverside Drive, Saint-Lambert, QC J4P 3P2, Canada

**Keywords:** saponin aqueous extract, green microwave-assisted hydrothermal, monoclinic copper oxide, cubic nickel oxide, magnetic properties, optical bandgap, PNP catalytic activity

## Abstract

In this study, we explored the formation of CuO nanoparticles, NiO nanoflakes, and CuO-NiO nanocomposites using saponin extract and a microwave-assisted hydrothermal method. Five green synthetic samples were prepared using aqueous saponin extract and a microwave-assisted hydrothermal procedure at 200 °C for 30 min. The samples were pristine copper oxide (100C), 75% copper oxide–25% nickel oxide (75C25N), 50% copper oxide–50% nickel oxide (50C50N), 25% copper oxide–75% nickel oxide (25C75N), and pristine nickel oxide (100N). The samples were characterized using FT-IR, XRD, XPS, SEM, and TEM. The XRD results showed that copper oxide and nickel oxide formed monoclinic and cubic phases, respectively. The morphology of the samples was useful and consisted of copper oxide nanoparticles and nickel oxide nanoflakes. XPS confirmed the +2 oxidation state of both the copper and nickel ions. Moreover, the optical bandgaps of copper oxide and nickel oxide were determined to be in the range of 1.29–1.6 eV and 3.36–3.63 eV, respectively, and the magnetic property studies showed that the synthesized samples exhibited ferromagnetic and superparamagnetic properties. In addition, the catalytic activity was tested against para-nitrophenol, demonstrating that the catalyst efficiency gradually improved in the presence of CuO. The highest rate constants were obtained for the 100C and 75C25N samples, with catalytic efficiencies of 98.7% and 78.2%, respectively, after 45 min.

## 1. Introduction

Even though more than three-quarters of the Earth’s surface is covered with water, only 2.5% of it is freshwater. The frozen water at the two poles represents approximately 70% of freshwater, and approximately 29% of this fresh water is locked in soil moisture or deep underground aquifers and is not directly usable by humans. Less than 1% of freshwater on Earth is easily accessible for human use. Securing a sufficient supply of pure water, in terms of both quantity and quality, is essential to human survival. However, this precious resource is becoming increasingly scarce, particularly in developing nations, owing to rapid development and industrialization [1,2,3].

Water pollution is a pressing global issue intensified by population growth and industrialization. Pollutants, such as dyes, heavy metals, and carcinogenic phenol derivatives, pose risks to aquatic life and the environment. Various water treatment techniques, including advanced oxidation processes (AOPs), photocatalysis, and adsorption, have been employed to address this challenge. AOPs offer rapid pollutant removal but can be costly and generate secondary pollution. Catalytic processes, particularly those involving common nanomaterials, have gained attention owing to their efficiency, affordability, and simplicity in wastewater treatment.

However, it is crucial to address the environmental concerns related to the use of toxic reactants and volatile chemicals in nanomaterial synthesis and subsequent processes. Consequently, there is a growing focus on green synthesis approaches that rely on substituting volatile organic solvents and other hazardous chemicals with safer alternatives in both the fabrication and capping processes of nanomaterials [1,2,3]. Green synthesis prioritizes the use of non-toxic and environmentally benign reagents, minimizes waste generation, and aims for energy efficiency in the production of various materials [4].

Green synthesis has been applied to preparing several types of nanoparticles (NPs), such as CuO, NiO, and CuO-NiO nanocomposites (NCs), using Capparis decidua leaf extract [5] and Psidium guajava leaf extract [6]. However, the construction of junctions between metal oxides with different bandgaps can improve their charge carrier separation and lifetime, thereby improving the catalytic characteristics and organic pollutant breakdown [7]. 

CuO-NiO nanocomposites are widely used as electrocatalysts for solid-state direct urea fuel cells [5], for methanol oxidation, in electrochemical sensors of dopamine [8], for their antibacterial activity [9], in photoconductive sensors [10], for the photocatalytic degradation of MB [11], for the reduction of toxic nitro pollutants [12], in pesticides [13], in supercapacitors [14], and in H_2_S sensors [15]. Various methods have been used to prepare CuO-NiO nanocomposites, including galvanic reactions [5], combustion [8], coprecipitation [9], surfactant-assisted precipitation [11], hydrothermal [14], microwave [14], green synthesis [13], ultrasonication [16], and sol-gel methods [17]. Microwave heating has several advantages, including the ability to directly heat materials quickly and selectively and achieve a uniform temperature throughout the reaction [18,19]. 

In this regard, L. Sui et al. prepared nickel oxide–copper oxide nanocomposites through deposition on a ceramic support by applying a hydrothermal treatment. They applied it to the sensing of hydrogen sulfide and found that this nanocomposite showed an enhanced response compared with NiO NPs [15]. P. Muhambihai et al. prepared several double oxides, such as ZnO-NiO, ZnO-CuO, and CuO-NiO, through simple precipitation with NaOH, followed by calcination [20]. They tested the photocatalytic efficiency of the calcined nanocomposites to decompose different dyes, and the best results were obtained using the nanocomposite NiO-CuO. The authors attributed this extraordinary behavior to the small particle size, reduced electron/hole pair combination, and increased *OH radical formation during degradation [20]. H. Lv et al. prepared copper oxide–nickel oxide nanocomposites by electrospinning nitrate precursors, followed by calcination, and applied them to the catalytic reduction of para-nitrophenol [21]. 

M. Kumar et al. prepared copper oxide–nickel oxide nanocomposites using a simple precipitation method [22]. They applied the prepared nanocomposites to an oxygen evolution reaction at a low potential of 0.15. V. L. Arun et al. prepared a nickel oxide–copper oxide nanocomposite through green synthesis using Azardiac indica leaf extract [7]. The use of NiO-CuO nanocomposites has been explored for their ability to catalyze the decomposition of MB and EY dyes, with catalytic degradation in the range of 78–99% [7]. E. F. Abo Zeid et al. prepared a nickel oxide–copper oxide nanocomposite through the decomposition of a bimetallic complex. The prepared composite was used to fabricate a chemical sensor to measure 2-aminophenol with a detection limit of 50 pM and a short response time of 14 s. [23]. V. Archana et al. prepared nickel oxide–copper oxide carbon nanocomposites through the decomposition of a metal organic framework, which were applied as electrochemical sensors of glucose [24].

In this work, we report for the first time the synthesis of CuO-NiO nanocomposites with different compositions using a microwave-assisted hydrothermal procedure in the presence of saponin extract. The effects of various CuO and NiO compositions on the characteristics of the prepared nanoparticles and nanocomposites were investigated using XRD, XPS, SEM, and TEM. Finally, the efficiencies of the nanoparticles and nanocomposites as catalysts for PNP were evaluated. 

## 2. Results and Discussion

### 2.1. FT-IR Characterization

**Figure 1:**
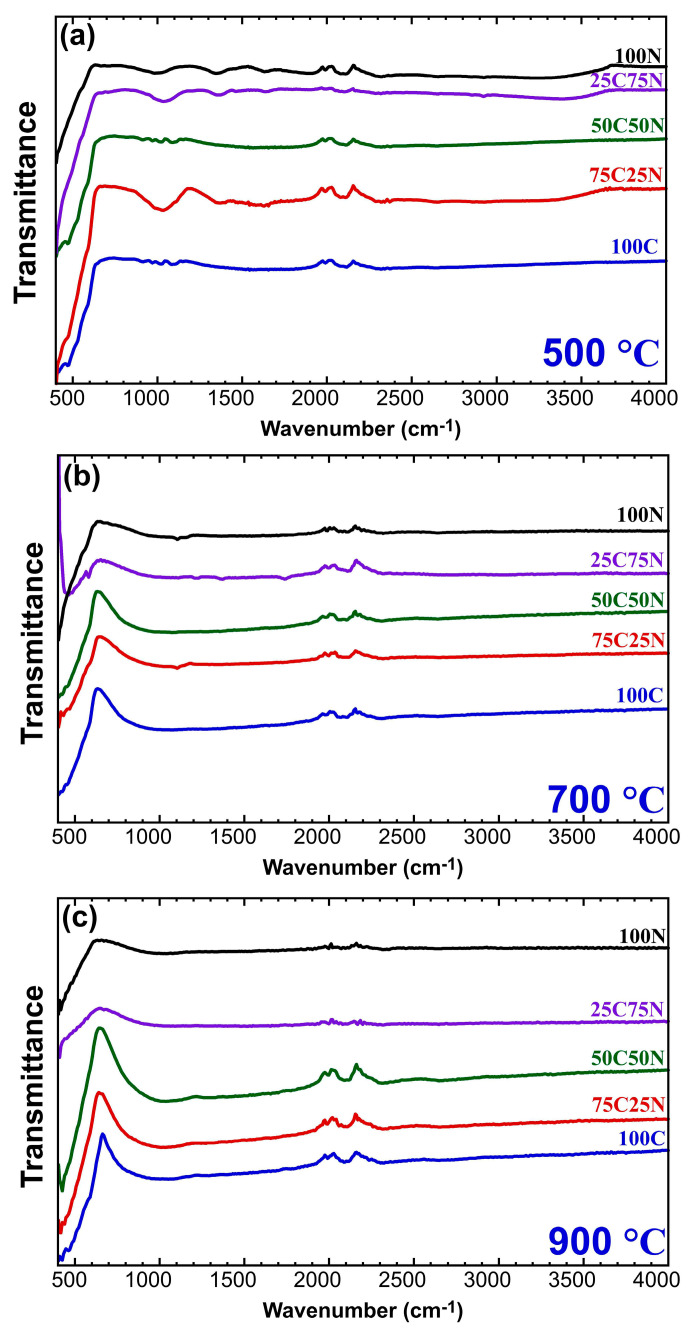
FTIR spectra of (**a**) 100C, 75C25N, 50C50N, 25C75N, and 100N calcined at 500 °C, (**b**) 700 °C, and (**c**) 900 °C.

To investigate the chemical composition and functional groups of the constituents of the composites, FT-IR analysis was performed on the calcined composites. Figure 1 shows the infrared spectra of the pristine CuO nanoparticles (100C) and pristine NiO nanoparticles (100N) and copper oxide–nickel oxide nanocomposites with different metal oxide ratios (75C25N, 50C50N, and 25C75N) calcined at three different temperatures: 500 °C, 700 °C, and 900 °C. Some patterns show a shallow band at ~3300 cm^−1^ for hydroxyl groups owing to the absorbed water molecules on the surface of some of the samples. Additionally, the band at ~2200 cm^−1^ was attributed to the C=C group in the remaining traces of the extract [25]. 

For some of the samples calcined at 500 °C, there was a band centered at ~1000 cm^−1^, which could be assigned to the bending of aromatic C-H or C-O-C remaining in the extract and disappeared when the samples were calcined at higher temperatures. The appearance of a broad band below 700 cm^−1^ can be attributed to Cu-O or Ni-O bonds. Sample 100C had bands at ~420 cm^−1^, ~507 cm^−1^, and ~609 cm^−1^ attributed to the Cu-O bond, and sample 100N exhibited peaks at ~430 cm^−1^ and ~575 cm^−1^ for the Ni-O bond. Varying the proportion of metal oxides within the composition of the composites is reflected in the change in the shape of the band in the 75C25N, 50C50N, and 25C75N samples. The FT-IR results confirm the formation of CuO-NiO composites, which is in accordance with the literature [25,26,27,28,29,30]. 

### 2.2. XRD Characterization

**Figure 2:**
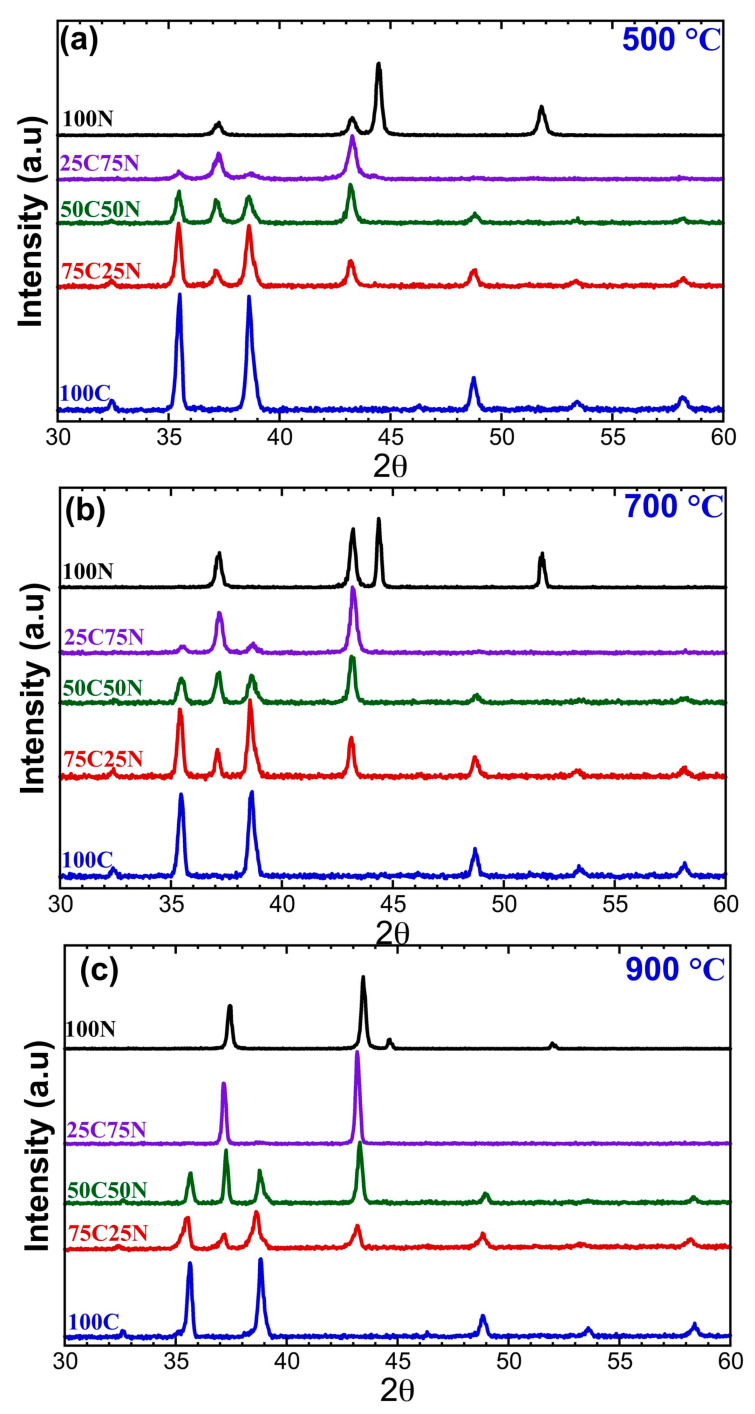
XRD diffractograms of (**a**) 100C, 75C25N, 50C50N, 25C75N, and 100N calcined at 500, (**b**) 700, and (**c**) 900 °C.

XRD analysis was performed on the calcined composites to investigate the crystallinity and phase identification of the constituents of the composites. Figure 2 shows the XRD diffractograms of the calcined pristine CuO and pristine NiO nanoparticles and CuO-NiO nanocomposites. Generally, all the samples containing nickel oxide showed peaks at 2θ = 37.26° and 43.21° for the (111) and (200) orientations of nickel oxide, respectively, which are related to the face-centered cubic phase according to JCPDS card 04-0835 [7,13,20,28,31]. In addition to the aforementioned peaks, sample 100N shows two extra peaks at 2θ = 44.48° and 51.84° for the (111) and (200) orientations of the Ni nanoparticles, respectively, according to JCPDS card 04-0850 [32,33]. It can be observed that as the calcination temperature increased from 500 °C to 900 °C, the Ni was oxidized into NiO. 

Copper oxide exhibits peaks at 32.5°, 35.5°, 38.71°, 48.81°, 53.39°, and 58.17° for the (110), (111), (202), (202), (020), and (202) orientations, respectively, which are related to the monoclinic phase according to JCPDS card 01-080-1268 [7,13,20,28,34]. Varying the proportion of metal oxides within the composition of the composites is reflected in the intensity of the peaks of both phases in the 75C25N, 50C50N, and 25C75N samples. The XRD results confirm the formation of CuO-NiO composites, which is in accordance with the literature [10,11,13,20,22,29].

The average crystallite sizes in the 100C, 75C25N, 50C50N, 25C75N, and 100N samples were 34, 31, 34, 36, and 34 nm, respectively. This increase in crystallite size can be attributed to the difference in radii between Ni^2+^ and Cu^2+^ ions. Although both ions have a positive charge, Ni^2+^ has a larger radius than Cu^2+^, which may contribute to the increase in the crystallite size. When copper oxide (CuO) was mixed with nickel oxide (NiO), the addition of NiO resulted in an increase in the bulk lattice of the resulting substance. The increase in sample 25C75N may not have followed the usual gradual progression, likely because of the saturation and accumulation of NiO at the grain boundaries of CuO, which caused the substitution process to cease. H. Weldekirstos et al. prepared a NiO-CuO nanocomposite using a CTAB-surfactant-assisted method, followed by calcination at 450 °C, and the average crystallite size was about 21 nm. The small crystallite size may be the result of using a high surfactant concentration (75 wt. %) for a metal precursor [11].

### 2.3. X-ray Photoelectron Spectroscopy (XPS) Analysis

**Figure 3:**
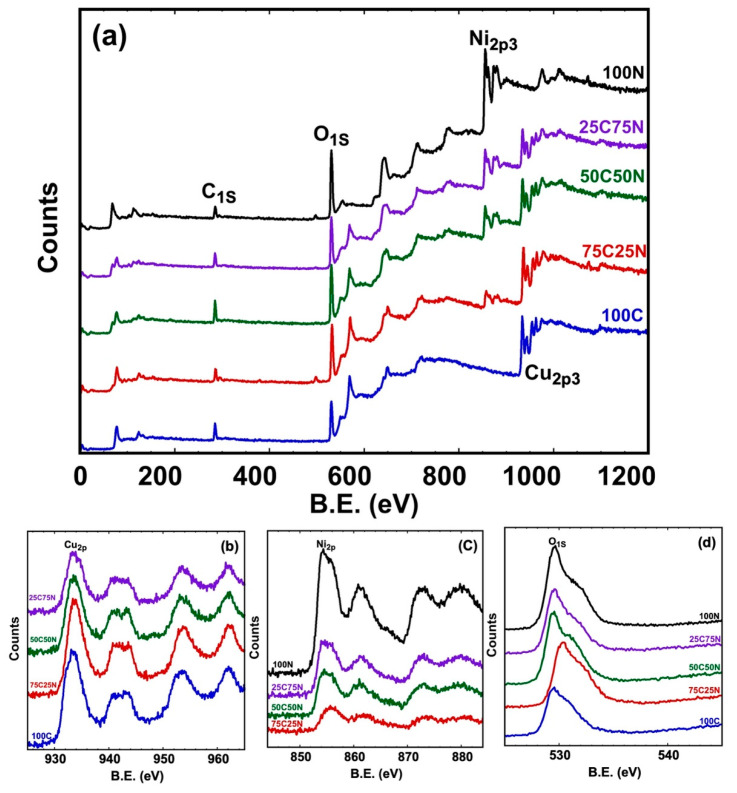
XPS spectra of 100C, 75C25N, 50C50N, 25C75N, and 100N: (**a**) survey spectra, (**b**) high-resolution XPS spectra of Cu2p, (**c**) high-resolution XPS spectra of Ni2p, and (**d**) high-resolution XPS spectra of O1s.

To investigate the elemental composition and oxidation states of the constituents of the composites, XPS analysis was performed on the calcined composites. Figure 3a shows the XPS survey spectra of 100C, 75C25N, 50C50N, 25C75N, and 100N, with peaks corresponding to Ni2p, Cu2p, O1s, and C1s observed. Figure 3b shows the high-resolution XPS spectra of Cu2p for samples 100C, 75C25N, 50C50N, and 25C75N. In these four samples, Cu2p is composed of doublet peaks at 933.27 eV for Cu2p_3/2_ and 953.59 eV for Cu2p_1/2_. In addition to this doublet, there are peaks at ~940.79 eV, 942 eV, and ~962 eV, which are known as satellite Cu2p peaks. The spin–orbit splitting value was approximately 20 eV, which is characteristic of the (+2) oxidation state of copper ions [26,35,36]. The intensity of the Cu2p doublet decreased with a decreasing copper oxide content in samples 100C, 75C25N, 50C50N, and 25C75N [37,38,39,40,41]. 

The high-resolution XPS spectra of Ni2p in the 75C25N, 50C50N, 25C75N, and 100N samples are shown in Figure 3c. The spectra show peaks at 854.38 eV and at 872.82 eV, which are assigned to Ni2p_3/2_ and Ni2p_1/2_. In addition to this doublet, there are peaks at ~860.84 eV and at ~879.81 eV, which are known as satellite Ni2p peaks. The spin–orbit splitting value was approximately 18.44 eV, which is characteristic of the (+2) oxidation state of Ni ions [7,16,19,22,42,43]. The intensity of the Ni2p doublet increased with an increasing nickel oxide content in the 75C25N, 50C50N, 25C25N, and 100N samples. Figure 3d shows the high-resolution XPS spectra of O1s in samples 100C, 75C25N, 50C50N, 25C75N, and 100N. The O1s peak appears at ~529 eV, which is attributed to the lattice oxygen (O^2−^) bound to the metal ions within the lattice, and a shoulder peak appears at ~532 eV due to the oxygen vacancies (V_o_) in the oxygen-deficient region [28,37,38]. The X-ray photoelectron spectroscopy (XPS) results show that the green microwave-assisted hydrothermal technique effectively creates NiO-CuO nanocomposites with either copper or nickel ions in a (+2) oxidation state, which does not change with a changing composition.

### 2.4. SEM and TEM Analyses 

**Figure 4:**
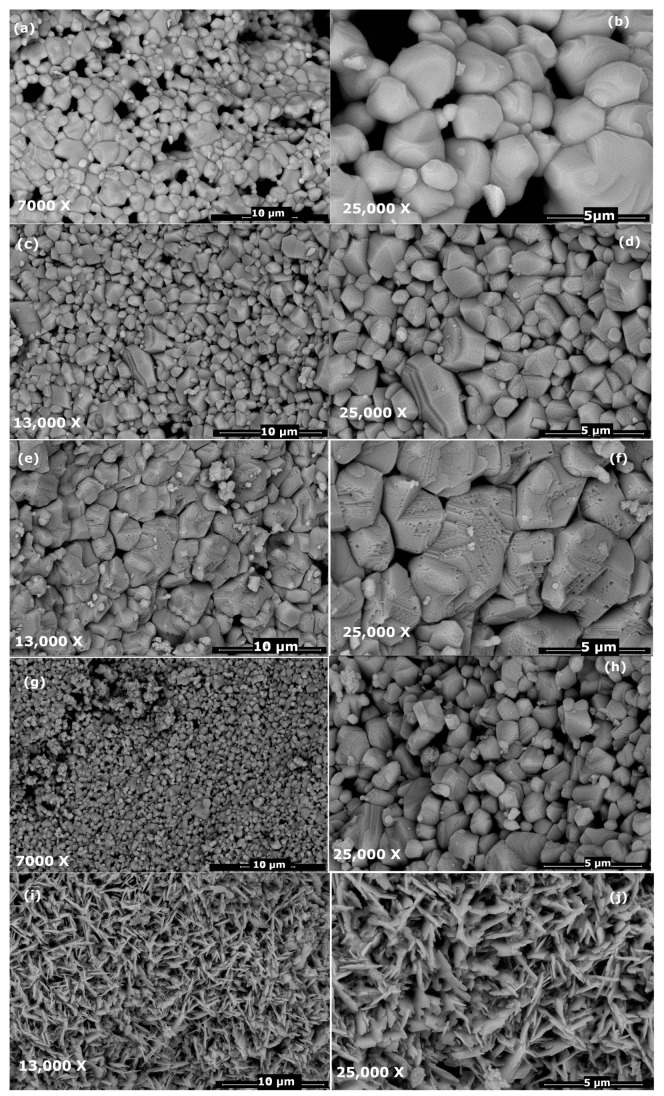
SEM images of (**a**,**b**) 100C, (**c**,**d**) 75C25N, (**e**,**f**) 50C50N, (**g**,**h**) 25C75N, and (**i**,**j**) 100N.

To investigate the surface morphology, including the size, shape, and distribution of the grains in the composites, SEM analysis was performed on the calcined composites. Figure 4a,b show the SEM images of sample 100C, which was a pristine copper oxide. It exhibited a polygonal structure with a homogenous size distribution. Figure 4i,j show the SEM images of sample 100N, which was a pristine nickel oxide. It exhibited a network-like structure composed of intersecting nanoflakes.

Substituting CuO with NiO in sample 75C25N resulted in a smaller grain size compared with sample 100C, as shown in Figure 4c,d. Increasing the NiO content in sample 50C50N (Figure 4e,f) resulted in stacked nanosheets, forming larger grains when compared with samples 100C or 75C25N. The further increase in NiO in sample 25C75N (Figure 4g,h) resulted in a smaller grain size than that in sample 50C50N. This may be attributed to the fact that the existence of added Ni^2+^ may destruct the formed nanosheets and consequently result in variation in the nanosheets’ construction, with smaller grains [15].

**Figure 5:**
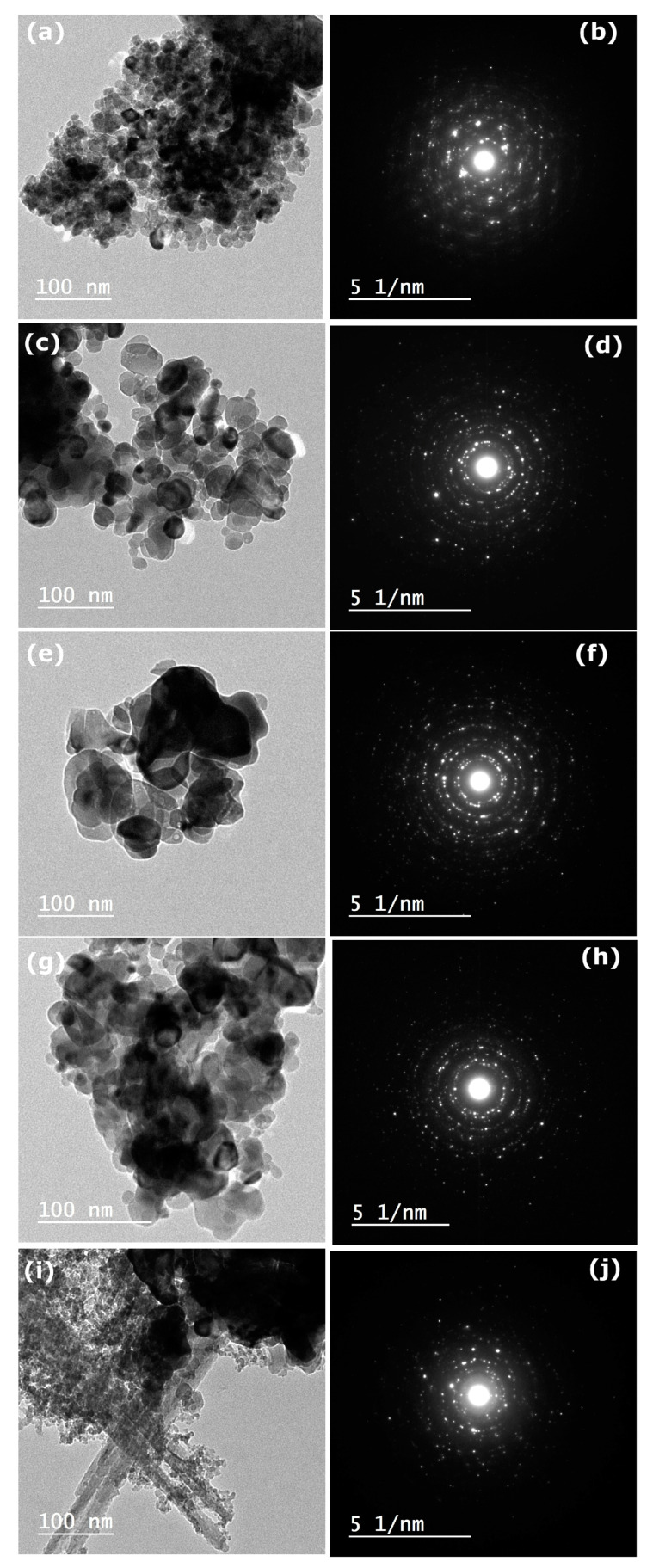
TEM images of (**a**,**b**) 100C, (**c**,**d**) 75C25N, (**e**,**f**) 50C50N, (**g**,**h**) 25C75N, and (**i**,**j**) 100N samples.

The TEM images in Figure 5 confirm the crystalline nature of the nanoparticles and nanocomposites, as can be seen in the SAED images. The average particle size of sample 100C was ~30.6 nm, which was composed of spherical nanoparticles (Figure 5a). The average particle size of sample 100 N (Figure 5i) was ~18.8 nm for the spherical nanoparticles, along with nanorods with lengths of ~800 nm and widths of ~20 nm. Substituting CuO with NiO in sample 75C25N (Figure 5c) resulted in an increase in the average particle size to 36.8 nm, and a further increase in nickel oxide in sample 50C50N (Figure 5e) resulted in an increase in the average particle size to 42.9 nm. On the other hand, a further increase in nickel oxide in sample 25C75N (Figure 5g) resulted in a decrease in the average particle size to 26.9 nm. 

The saponin extract was used as a capping agent to prepare the CuO-NiO nanocomposites. The suggested mechanism of metal oxide nanocomposite formation may be described as follows. Nickel nitrate and copper nitrate were decomposed through hydrolysis to form hydrated metal species, which were precipitated by adding KOH to form metal hydroxides. The metal hydroxide precipitate was transferred into an autoclave and subjected to microwave hydrothermal treatment (MHT). The temperature of the MHT can accelerate the nucleation and growth of nanoparticles. Once the nuclei are formed, the dissolved metal species continue to deposit onto the existing nuclei, leading to the growth of NiO-CuO nanocomposites. The green extract, which can act as a stabilizing agent, may introduce organic functional groups onto the surface of the nanoparticles. 

### 2.5. UV-Vis Diffuse Reflectance Spectroscopy

**Figure 6:**
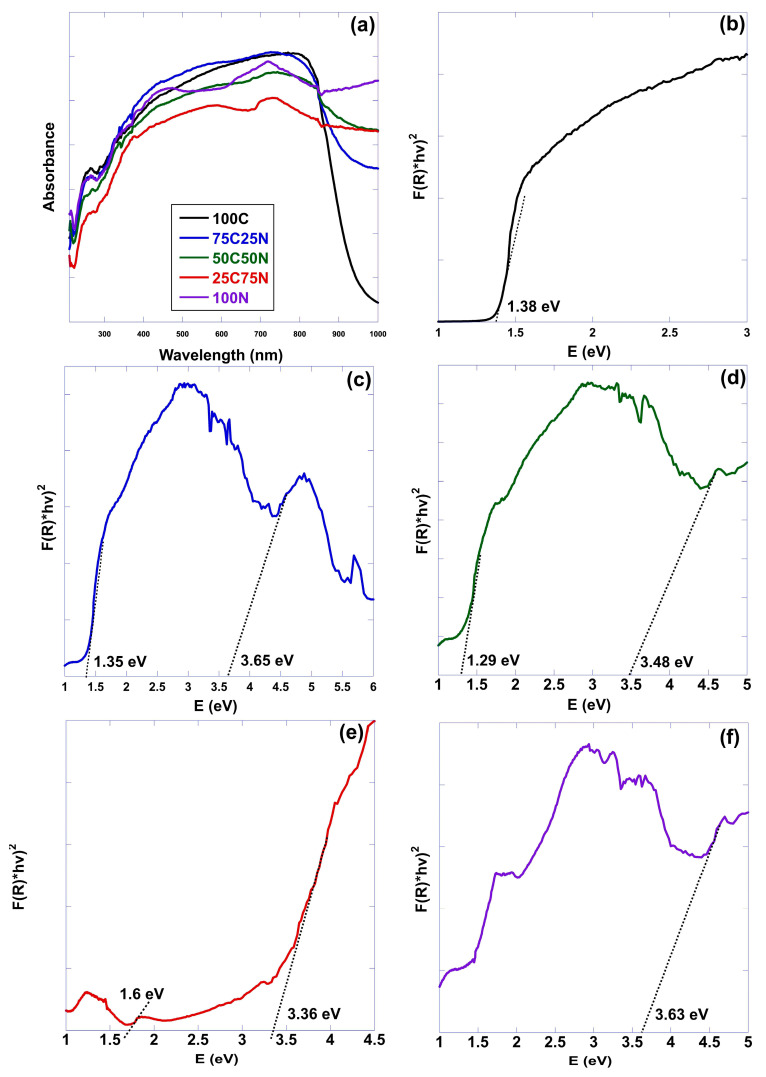
(**a**) Diffuse reflection spectra of samples, corresponding Kubelka–Munk results for (**b**) 100C, (**c**) 75C25N, (**d**) 50C50N, (**e**) 25C75N, and (**f**) 100N.

UV-visible diffuse reflectance spectroscopy was used to investigate the optical properties of the pristine CuO and NiO nanoparticles and CuO-NiO nanocomposites. The Kubelka–Munk model was used to estimate the optical bandgaps (Eg). The results are presented in Figure 6, where (hν) is plotted on the *x*-axis and (∝hν)^2^ is plotted on the *y*-axis. 

Figure 6a shows the DRS spectra of all the samples. The samples exhibited a wide absorption band ranging from 400 nm to 800 nm [44]. Samples 100C and 75C25N had higher absorption intensities, indicating that they can absorb more photons and, in turn, enhance the photocatalytic activity [45]. The calculated optical bandgaps of pristine CuO and NiO were 1.38 eV and 3.63 eV, respectively, which are comparable to the values reported in the literature (i.e., 1.2–1.5 eV for CuO and 3.6–4 eV for NiO) [7,17,46,47].

The CuO-NiO nanocomposites exhibited two optical bandgaps. The optical bandgap values of CuO in the nanocomposites were 1.35 eV, 1.29 eV, and 1.6 eV in 75C25N, 50C50N, and 25C75N, respectively. The optical bandgaps of NiO in the nanocomposites were 3.36 eV, 3.48 eV, and 3.65 eV in 25C75N, 50C50N, and 75C25N, respectively. It can be observed that the optical bandgap varies with the composition of the metal oxides, which can be attributed to the mixing phase effects and the creation of new energy levels. 

S. Senobari et al. prepared a CuO-NiO nanocomposite with an equal molar ratio using a sol-gel method and reported optical bandgap values of 1.57 eV and 3.50 eV for CuO and NiO [17]. L. Arun et al. synthesized a CuO-NiO nanocomposite using a green method with the leaf extract of Azardica indica with molar ratios of the corresponding oxides of 3:1, 1:1, and 1:3 (i.e., 3C1N, 1C1N, and 1C3N). Two optical bandgaps of 1.89 eV, 1.84 eV, and 1.98 eV for CuO and 3 eV, 2.8 eV, and 3.4 eV for NiO were seen in samples 3C1N, 1C1N, and 1C3N, respectively [7]. They attributed the nonlinear change in the bandgap with the ratio of metal oxides to the exchange interaction between the sp-d orbitals. N. Bayal et al. prepared CuO-NiO nanocomposites with equal molar ratios. They reported optical bandgap values for pristine NiO and pristine CuO of 4.28 eV and 2.1 eV, respectively. The reported bandgap values for NiO and CuO composites were 4.17 eV and 1.9 eV, respectively [47].

### 2.6. Magnetic Properties

**Figure 7:**
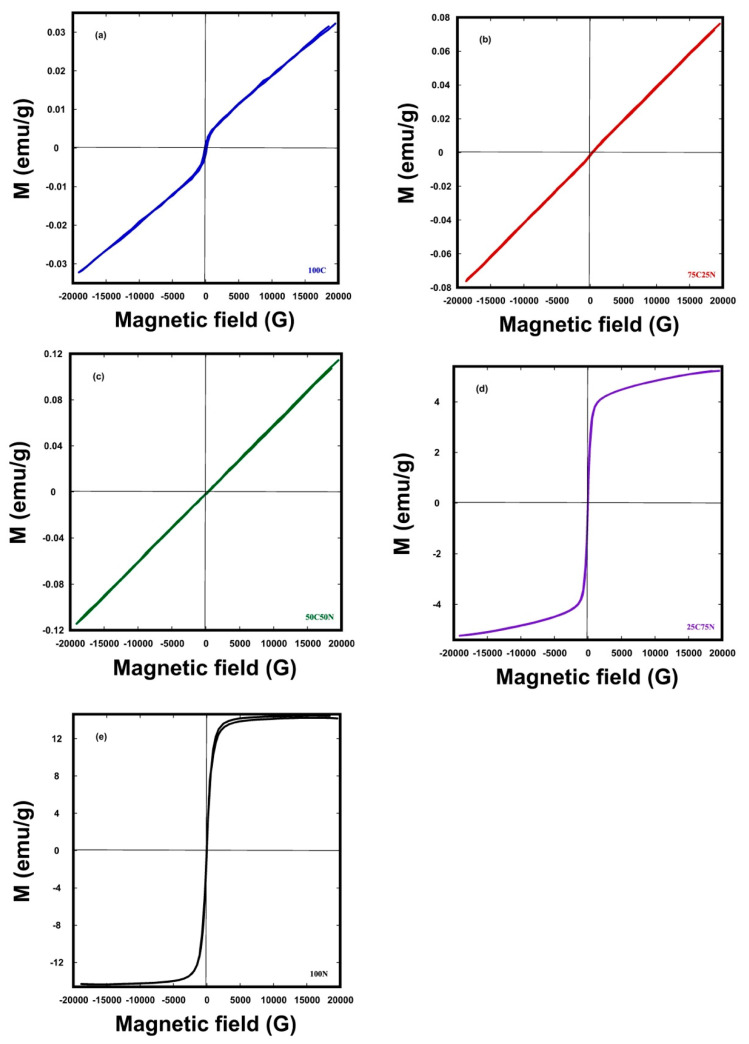
M-H curves of (**a**) 100C, (**b**) 75C25N, (**c**) 50C50N, (**d**) 25C75N, and (**e**) 100N.

Figure 7 shows the M-H curves recorded at room temperature using a vibrating sample magnetometer (VSM) with an applied field of 20 kOe for the nanoparticles and nanocomposites. An M-H curve is a graphical representation of the relationship between the magnetization of a material and the magnetic field applied to it. As the magnetic field increases in the positive direction from zero, the magnetization of the material also increases. This initial increase is due to the alignment of the material’s individual magnetic moments or spins with the direction of the applied field. At a particular point, most of the magnetic moments are aligned with the applied field, and the material reaches its maximum magnetization, which is referred to as saturation magnetization. This is indicated by the leveling-off of the curve. When the magnetic field starts to decrease, if the magnetization returns entirely to zero and no hysteresis is formed, then the material is paramagnetic, as a random orientation returns upon the removal of the field. However, when the magnetic field starts to decrease, and if the magnetization does not return entirely to zero and hysteresis is formed, then the material is ferromagnetic. This is because some magnetic moments remain aligned because of the intrinsic properties of the material. This residual magnetization is referred to as remanence. The coercive field is the amount of reverse magnetic field required to bring the magnetization back to zero. This indicates the difficulty of demagnetizing the material. A larger coercive field area signifies a greater hysteresis and a higher energy requirement to demagnetize the material.

It has been reported that CuO in bulk form and as nanoparticles shows ferromagnetism due to the uncompensated surface spin of copper ions [7,30,48,49]. NiO exhibits antiferromagnetism in its bulk form and ferromagnetism as nanoparticles [19,30,48,49]. The weak ferromagnetism recorded in NiO is attributed to vacancies [19]. The samples prepared in this study exhibited various magnetic behaviors based on their compositions. Sample 100C shows weak ferromagnetism with remnant magnetization (Mr) and a coercivity (Hc) of 2.89 × 10^−3^ emu/g and 0.275 kOe, respectively. Sample 100N shows mixed behavior: superparamagnetic-like behavior at a high magnetic field and weak ferromagnetic-like behavior at a low magnetic field. The same behavior was observed for sample 25C75N. The remnant magnetization (Mr) and coercivity (Hc) were 0.398 emu/g and 0.021 kOe for sample 100N and 0.215 emu/g and 0.017 kOe for sample 25C75N. In contrast, 75C25N and 50C50N exhibited paramagnetic behavior.

### 2.7. PNP Catalytic Reduction 

**Figure 8:**
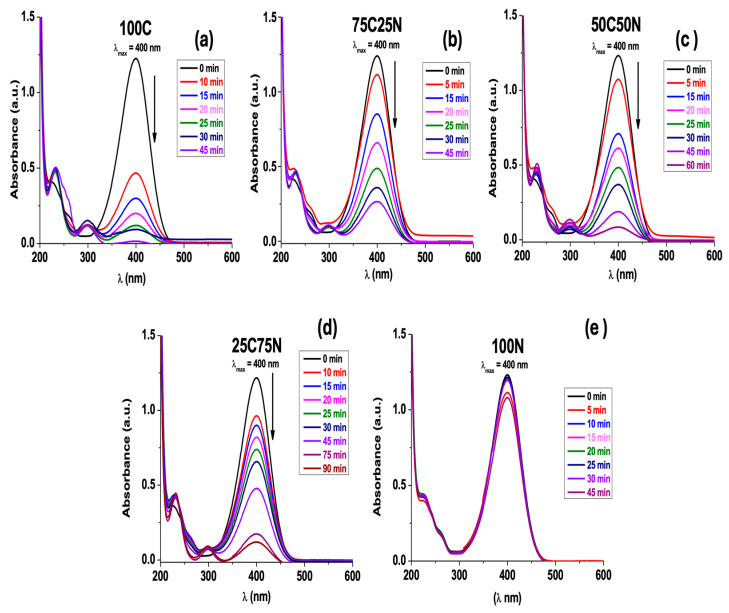
UV-Vis spectra of PNP’s reduction by NaBH_4_ in presence of catalysts (**a**) 100C, (**b**) 75C25N, (**c**) 50C50N, (**d**) 25C75N, and (**e**) 100N.

The para-nitrophenol (PNP)-reducing catalytic activity of the prepared materials was examined at room temperature by recording the UV-Vis absorption of PNP versus time in the presence of sodium borohydride (NaBH_4_) in the range of 200–600 nm (Figure 8). The reduction of PNP occurred owing to the auto-hydrolysis of NaBH_4_ and thus the generation of hydrogen, as described in the following equation:

NaBH_4_ (aq) + 2H_2_O (l) → NaBO_2_ (aq) + 4H_2_(g)

**Figure 9:**
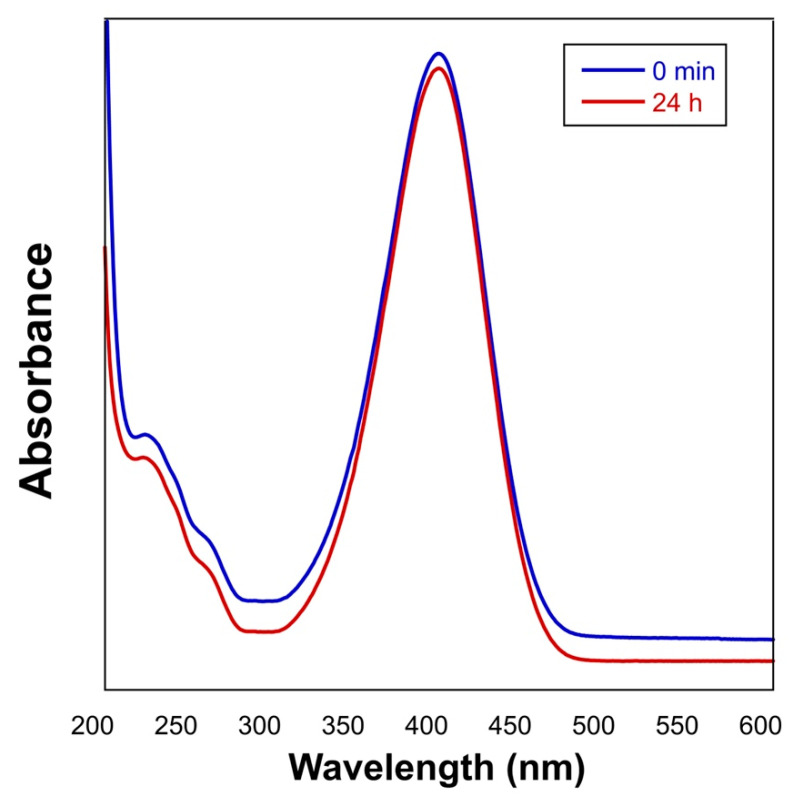
UV-Vis spectra of PNP’s reduction by NaBH_4_ in the absence of a catalyst.

In the absence of a catalyst, the reduction of PNP was very slow, and its conversion into AP was negligible after 24 h (Figure 9). However, in the presence of the catalysts, reduction occurred rapidly at room temperature, as shown in Figure 8.

It is well known that the reduction of nitrophenols in the presence of excess NaBH_4_ proceeds via the formation of phenolate ions, which are then transformed into amino-phenols. Thus, the peak centered at 400 nm was attributed to phenolate ions. The intensity of this peak decreased (Figure 8) as the reduction proceeded, leading to the formation of the amino phenol product. 

For each experiment, the rate constant of the reduction process was determined from the time-dependent decrease in the intensity of the absorption peak (A) at 400 nm. The reaction proceeded according to a pseudo-first-order kinetic reaction because the concentration of sodium borohydride can be considered constant. The apparent rate constants were calculated directly from the linear relationship between ln(A/A0) and time, where A0 is the initial absorbance intensity of PNP at 400 nm.

**Figure 10:**
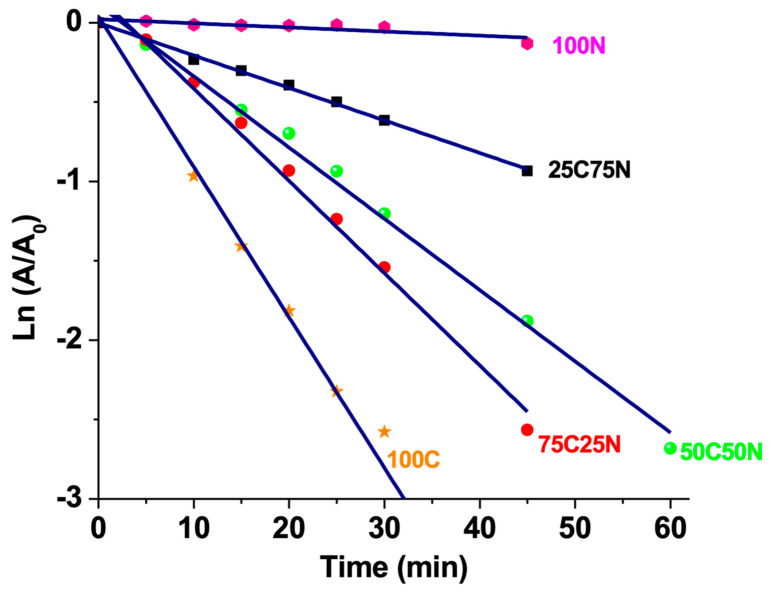
Rate of PNP’s reduction by NaBH_4_ in the presence of different catalysts.

Kinetic plots of the PNP reduction processes in the presence of different catalysts are shown in Figure 10. The apparent rate constants (k_app_, min^−1^) were determined from linear kinetic plots. The rate constant k_app_ (min^−1^) values of the PNP reduction were 2.59 × 10^−3^ min^−1^, 2.05 × 10^−2^ min^−1^, 4.49 × 10^−2^ min^−1^, 5.83 × 10^−2^ min^−1^, and 9.48 × 10^−2^ min^−1^ for samples 100N, 25C75N, 50C50N, 75C25N, and 100C, respectively.

Pure nickel oxide (100N) exhibited the slowest activity, followed by 25C75N, 50C50N, 75C25N, and 100C. Indeed, copper oxide (CuO) exhibits the ability to readily transition between the oxidation states of Cu(I) and Cu(II), thereby offering greater potential for electron transfer. In contrast, nickel oxide (NiO) primarily exists in the Ni(II) state, limiting its participation in certain redox reactions. CuO possesses a crystal structure that allows for the formation of more oxygen vacancies than NiO. These vacancies served as active sites for the adsorption and activation of reactant molecules, thereby enhancing the catalytic activity of CuO. Moreover, CuO typically possesses a smaller bandgap than NiO, which facilitates easier excitation of electrons into the conduction band, thereby increasing their involvement in catalytic cycles [50,51]. The catalytic efficiencies after 45 min for 100 N, 75C25N, and 100 C were 12.2%, 78.2%, and 98.7%, respectively.

The samples used in this study showed a better catalytic activity than those reported in the literature. For example, CuO prepared using microwave heating showed a rate constant of 2.2 × 10^−2^ min^−1^ [52]. NiO and CuO prepared via Capparis decidua green synthesis showed rate constants of 8.4 × 10^−3^ min^−1^ and 3.3 × 10^−2^ min^−1^, respectively.

## 3. Materials and Methods

### 3.1. Materials

Nickel nitrate hexahydrate (Ni(NO_3_)_2_·6H_2_O), copper nitrate hexahydrate (Cu(NO_3_)_2_·6H_2_O), and ammonium solution 30% were purchased from Sigma-Aldrich, St. Louis, MO, USA. The primary chemicals used in this study were of the highest quality and analytical-grade. Double-distilled water was used to prepare all the solutions. The soapnuts were sourced from NaturOli Beautiful in Peoria, AZ, USA, and were sun-dried, de-seeded, and certified as organic by the USDA.

### 3.2. Saponin Extraction

A household grinder was used to grind the soapnuts into a fine powder. A mixture of soapnut powder and deionized water was prepared by combining 1 g of soapnut powder with 10 mL of deionized water. The mixture was magnetically stirred for 1 h at 60 °C. Whatman filter paper was used to isolate the extract, which was then chilled at 4 °C in a refrigerator. The filtrate was then used and labeled as the saponin extract. 

### 3.3. Microwave-Assisted Hydrothermal Synthesis

**Scheme 1:**
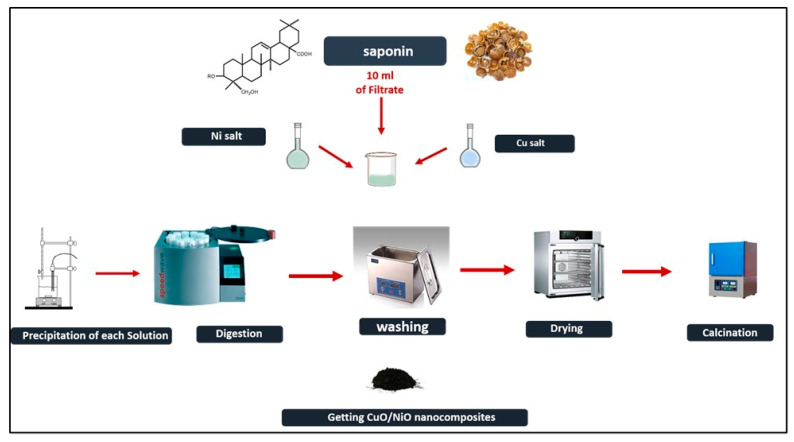
Synthesis of CuO-NiO nanocomposites.

To prepare a 0.1 M concentration stock solution for each salt, weighed amounts of nickel salt and copper salt were used. The names and formulations of these samples are listed in Table 1. For example, 50 mL of nickel stock solution was mixed with 50 mL of copper stock solution, and 20 mL of the extract was added. Continuous stirring was employed to precipitate the material while adding NH_4_OH solution dropwise at a rate of 1 mL/min using a burette. The pH of the solution was monitored using an Orion 2-Star pH meter until it reached a pH of 10. The synthetic process is illustrated in Scheme 1. The hydroxide precipitate prepared in this step was transferred into a Teflon vessel and then heated in a microwave at 200 °C for 30 min [53]. The synthetic conditions were selected based on a previous study [53].

The sample was subsequently removed from the microwave and the liquor poured into a beaker. A Powersonic 405 system was used to sonicate the sample for 90 min in 50 mL of distilled water. After 12 h of exposure to gravity, a solid precipitate was formed, and the supernatant was removed and replaced with 20 mL of ethanol. Sonication and precipitation were carried out multiple times, followed by drying the sample at 100 °C for 12 h in an oven. The samples were calcined at three different temperatures (500, 700, and 900 °C) for a period of two hours.

### 3.4. Powder Characterization

The FT-IR spectra of the metal oxide nanoparticles and nanocomposite samples were obtained using a Cary 630 FT-IR spectrophotometer. The crystalline phases of the metal oxide nanocomposites were determined using XRD analysis using a Bruker D8 X-ray diffractometer with Ni-filtered Cu-Kα radiation and a graphite monochromator. The X-rays were produced with a wavelength of 1.54060 Å at 35 kV and 25 mA, a glancing angle ranging from 10° to 60°, and scan steps of 0.02°. The accuracy of the analysis was <0.001°.The Scherrer formula was used to determine the crystallite size based on the half width of the maxima present in the XRD peaks. Scanning electron microscopy (SEM) (FE-SEM, QUANTA FEG 250, FEI, Netherlands -Operated at 20 kv) was used to determine the surface morphology. This scanning electron microscope had an accelerating voltage of 30 kV and a magnification capability of up to 400,000×. To conduct the TEM imaging, a JEOL JEM-1011 transmission electron microscope with a high resolution was used.

A Thermo Scientific Evolution 300 UV–visible spectrophotometer was used to measure the diffuse reflectance UV–visible spectra (DRS). The reflectance data were converted into absorbance, and the optical bandgaps of the calcined samples were determined using the Kubelka–Munk Equation (1).

(1) $$\propto \;=F\left(R\right)=\frac{{\left(1-R\right)}^{2}}{2R}$$

F(R) is the Kubelka–Munk function, which corresponds to the absorbance; R is the reflectance; h is the blank constant; and ν is the frequency [54]. The bandgap value can be calculated by extrapolating the linear part of a graph, where (F(R)h*ν*)^2^ is plotted on the *y*-axis against h*ν* on the *x*-axis [55].

X-ray photoelectron spectroscopy was performed using a K-Alpha instrument (Thermo Fisher Scientific, Waltham, MA, USA) to analyze the surface and determine the oxidation state. The experiment used monochromatic X-ray Al K-alpha radiation ranging from −10 to 1350 eV, with a spot size of 400 and a pressure of 10^−9^ mbar. The full-spectrum pass energy was set to 200 eV, and the narrow spectrum was set to 50 eV. The charge correction was based on the binding energy of the C1s peak at 284.4 eV. To assess the magnetic properties, measurements were conducted using a Lake Shore VSM 7410 model, which is a 3 T magnet. A total of 0.02 gm of NaHBO_4_ was added to 50 mL of PNP solution (10 ppm concentration), and then 0.02 gm of each sample was added after activation at 120 °C for 30 min. The solution was shaken at 250 rpm. The change in the concentration of PNP was determined using a Cary Series UV-Vis spectrophotometer (Agilent Technologies, Santa Clara, CA, USA).

## 4. Conclusions

In this study, we report a novel method for the preparation of CuO-NiO nanocomposites using microwave-assisted hydrothermal green synthesis. This technique is eco-friendly and efficient in terms of time and energy consumption. The composition of the nanocomposite was varied by changing the ratio of the Cu and Ni precursors during synthesis. The samples were characterized using techniques such as FT-IR, XRD, XPS, SEM, and TEM. The samples contained cubic nickel oxide and monoclinic copper oxide in the +2 oxidation state. The optical bandgap of copper oxide was found to be in the range of 1.29–1.6 eV, while the optical bandgap of nickel oxide was in the range of 3.36–3.63 eV. Magnetic property studies revealed that the synthesized samples exhibited ferromagnetic and superparamagnetic properties. The catalytic activity was tested against para-nitrophenol, and the results showed that the catalyst efficiency increased in the presence of CuO. The highest rate constants were obtained for the 100C and 75C25N samples, with catalytic efficiencies of 98.7% and 78.2%, respectively, after 45 min. This study allowed us to establish a correlation between the composition and structure of the nanocomposites and their catalytic activities. This study paves the way for the development of efficient ecofriendly synthesized materials for various environmental applications.

## Figures and Tables

**Table 1:** Sample names and corresponding copper–nickel volumes.

Sample Code	Vol. of Copper Salt [0.1 M] (mL)	Vol. of Nickel Salt [0.1 M] (mL)
100C	100	-
75C25N	75	25
50C50N	50	50
25C75N	25	75
100N	-	100

## Data Availability

Data are available only upon request from the corresponding authors.
